# Navigating uncertainty together: a participatory mixed-method study of counseling services for couples living with multiple sclerosis

**DOI:** 10.3389/fpubh.2026.1816630

**Published:** 2026-06-24

**Authors:** Jörn Nielsen, Theresia Krieger, Elke Kalbe, Ann-Kristin Folkerts

**Affiliations:** 1 Department of Medical Psychology, Neuropsychology and Gender Studies and Center for Neuropsychological Diagnostics and Intervention (CeNDI), Faculty of Medicine and University Hospital Cologne, University of Cologne, Cologne, Germany; 2 Department of Cognitive Rehabilitation, Johanniter Clinic Godeshöhe GmbH, Bonn, Germany; 3 Department of Applied Social Sciences, Hochschule Niederrhein, Campus Mönchengladbach, Mönchengladbach, Germany; 4 partieval—Advancing Participatory Skills, Process Support, and Evaluation in Health (GmbH), Aachen, Germany

**Keywords:** communication, community-based participatory research, counseling, couples, focus groups, multiple sclerosis, qualitative research, expert interview

## Abstract

**Introduction:**

Multiple sclerosis (MS) is a progressive neurological disease that affects not only individuals diagnosed but also their partners and relationships. However, couple-based counseling services and couples’ preferences regarding such interventions remain insufficiently studied. The PAART study addressed this gap by exploring the counseling needs of couples living with MS and identifying suitable approaches for outpatient counseling services.

**Methods:**

This study applied a participatory health research (PHR) approach involving researchers, practitioners, and people living with MS as co-researchers throughout the project. A sequential qualitative design was conducted in two stages: first, expert interviews (EIs) with counselors from German MS Society service centers; second, virtual focus group discussions (FGDs) with couples living with MS. Findings from the interviews informed the focus of the FGDs. Data were analyzed using qualitative deductive–inductive content analysis based on the participatory DEPICT method.

**Results:**

Ten counselors participated in the EIs, and four FGDs were conducted with 16 couples recruited nationwide. Both groups emphasized the importance of couple-oriented counseling, particularly around the time of diagnosis, with relationship-related concerns often outweighing disease-specific topics. Communication difficulties and balancing individual and shared needs emerged as central challenges. Counselors additionally highlighted caregiver burden, personal boundaries, and shame. Across both groups, uncertainty about the future was identified as a key theme.

**Conclusion:**

Findings from the EIs and FGDs were highly consistent, underscoring the importance of relationship-focused support in addressing present- and future-oriented challenges associated with MS. The results suggest that healthcare professionals should be prepared to address couple dynamics sensitively and effectively. Integrating couple-oriented counseling into outpatient psychosocial and rehabilitation services may strengthen relationship resilience, improve shared coping strategies, and support the long-term well-being of both partners.

**Clinical trial registration:**

https://www.drks.de/DRKS00031739, Identifier DRKS00031739.

## 1 Introduction

Multiple sclerosis (MS) is a chronic inflammatory and degenerative disorder of the central nervous system characterized by heterogeneous motor and nonmotor symptoms that can substantially impair functioning and quality of life (1). Global MS prevalence has increased steadily in recent years, with approximately 2.8 million people affected worldwide and around 280,000 living with MS in Germany (2). Because MS commonly manifests between the ages of 20 and 40 years, the disease often coincides with major life transitions such as establishing partnerships, starting families, and developing careers (3).

Beyond its physical consequences, MS can place considerable strain on intimate relationships. People with MS (pwMS) frequently experience depression, cognitive changes, and reduced participation in everyday activities due to fatigue (4). Partners may also face significant emotional and caregiving burdens, with studies reporting elevated levels of psychological distress and depressive symptoms among partners of pwMS (5, 6). Relationship quality appears to play a central role in coping and adaptation, as stronger partnerships are associated with better illness acceptance and psychological well-being (7–10). At the same time, MS-related stressors and socioeconomic consequences may contribute to relationship instability and separation (6, 11, 12).

Research adopting a dyadic perspective suggests that supportive couple dynamics may improve stress management, partnership satisfaction, and psychosocial adjustment in couples living with MS (13, 14). However, existing evidence remains limited. Previous studies have primarily relied on correlational designs and often focus on the caregiving partner rather than the couple as a relational unit (13). Moreover, couple-based psychosocial interventions for MS remain scarce and insufficiently evaluated (15–17).

To better understand the current state of research, we previously conducted a systematic review of dyadic psychosocial interventions for couples living with MS (18). Of 2,878 identified records, only eight studies met the inclusion criteria. While these studies suggested potential benefits for relationship quality, symptom management (particularly sexual dysfunction), and emotional well-being, they also revealed substantial methodological heterogeneity and a lack of evidence regarding which counseling themes and formats are most relevant and acceptable for couples. Only one study explored participants’ perspectives on intervention content through qualitative interviews (19), highlighting the need for further practice-oriented and context-sensitive research, particularly within the German healthcare system.

The present PAART study (derived from Paar, the German word for “couple,” and “participation”) builds on these findings by focusing explicitly on the counseling needs and preferences of couples living with MS and on the practical design of outpatient counseling services. To achieve this, we adopted a participatory health research (PHR) approach involving researchers, practitioners, and people living with MS as co-researchers throughout the project. This approach reflects contemporary patient-centered research principles and aims to ensure that the study design, data interpretation, and resulting recommendations are closely aligned with the lived experiences and practical needs of affected couples (20, 21). This approach is consistent with patient engagement frameworks which are gaining importance in medical and health research (22) and reflects Charlton’s principle of “nothing about us without us” (23).

The overarching research questions of the PAART study were: What are the counseling needs of couples living with MS, and how can an outpatient counseling model be appropriately designed regarding content and structure?

## 2 Materials and methods

### 2.1 Research approach

The PAART study was guided by a participatory health research (PHR) approach, which conceptualizes research as a collaborative process involving relevant healthcare stakeholders (24). In line with this approach, persons and partners living with MS and counselors from German MS Society service centers were actively involved throughout the study process. This participatory framework was chosen to ensure that the study remained closely grounded in the lived experiences and practical support needs of couples affected by MS.

### 2.2 Qualitative study design

**Figure 1:**
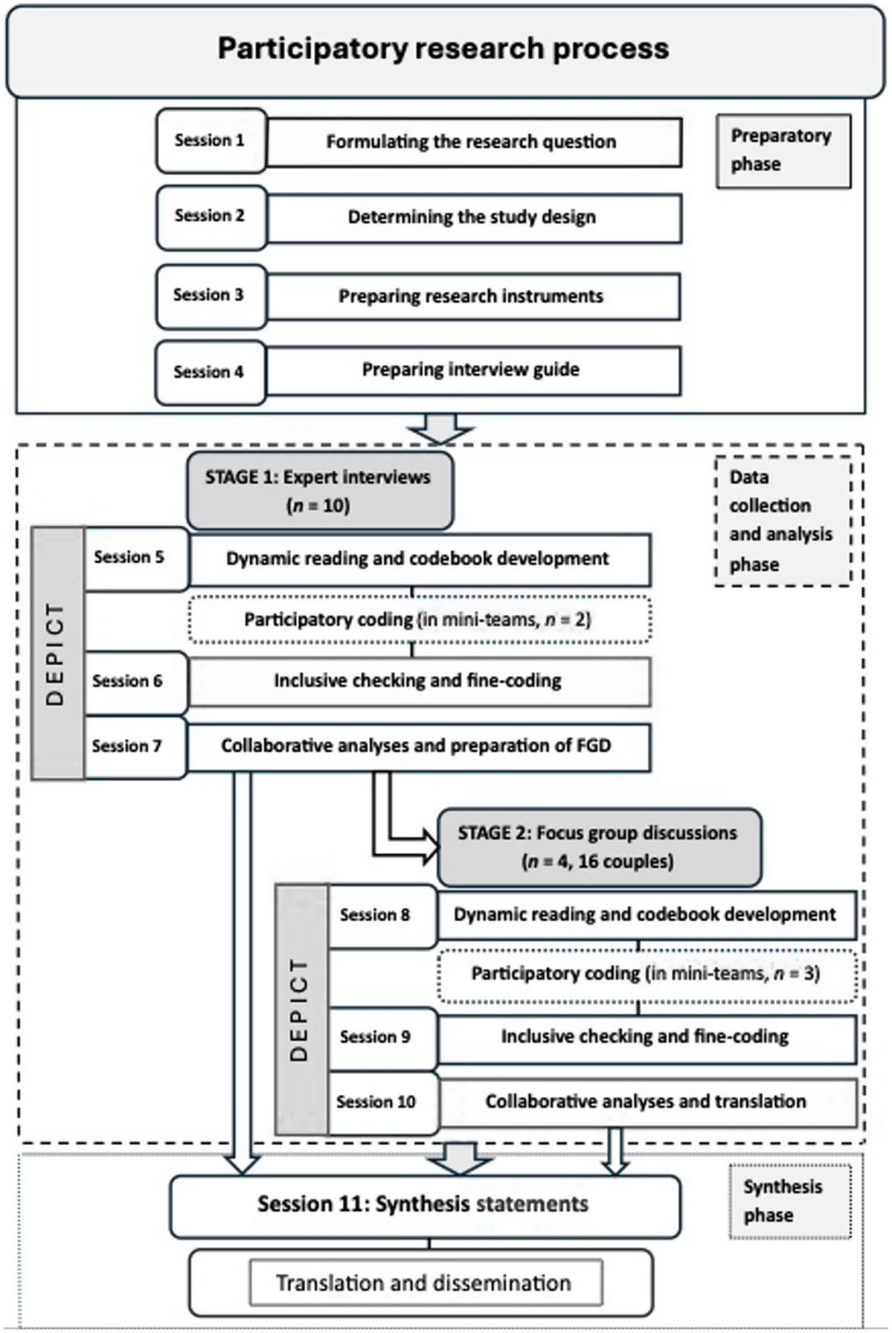
Overview of the participatory process throughout the entire PAART study.

The PAART study employed an exploratory QUAL–qual sequential mixed-methods design (25–27), comprising two qualitative data collection phases of equal priority. Expert interviews (EIs) with counselors from the German Multiple Sclerosis Society generated initial insights that informed the design of the subsequent focus group discussions (FGDs) with couples living with MS. This sequential approach was chosen to inform and validate the interview guide for the FGDs. Both qualitative components generated independent findings, which were subsequently integrated to address the central research questions. An overview of the overall study design is presented in Figure 1. The study was conducted and reported in accordance with the Standards for Reporting Qualitative Research (SRQR) (28).

### 2.3 The PAART research team

Following PHR principles, a co-researcher team was assembled at the outset. The team comprised two pwMS, two partners of pwMS (*n* = 4), and six experts from clinical and outpatient counseling settings (*n* = 6), including an MS nurse, a neurologist, a psychologist, and three counselors from the German MS Society representing the municipal, state, and national levels.

The co-researchers actively shaped the research process in collaboration with two academic researchers (JN, AKF) (*n* = 2), forming the overall PAART research team. The PAART research team was externally supported by an expert in PHR approaches (TK) and a so-called “critical friend” (EK) who provided reflective critique throughout the project. All team members were recruited by the project initiator (JN), with most suggestions for team members made by the participants themselves. As part of the participation request, the co-researchers were introduced to the core idea and encouraged to help shape the project, as diversity in perspectives was regarded as essential. The co-researchers contributed voluntarily and were not financially compensated.

All PAART research team meetings were conducted via video conference for efficiency and practicality and lasted between 60 and 90 min.

### 2.4 Ethical considerations

The PAART study was approved by the Ethics Committee of the Medical Faculty of the University of Bonn (reference number AZ 153/22; June 9, 2022). All members of the co-researcher team signed a “code of trust” to ensure the confidential handling of all collected data. This procedure was communicated to the study participants, and the names and affiliations of all team members were listed at the end of the study information sheet. All participants provided written informed consent prior to participation. Interview material which could potentially identify interviewees was anonymized as required (e.g., redacting references to specific locations).

### 2.5 Participatory study process

The PAART research team held its constitutive meeting on February 21, 2022 (see Figure 1 for an overview of the entire participatory research process). During this first session, the project initiator (JN) introduced the principles of the PHR approach and outlined the research problem based on relevant literature. Participants shared practical experiences, jointly formulated the central research questions, discussed suitable methods and instruments, and considered initial recruitment strategies. During Sessions 2–4, the team developed the study design, defined the inclusion and exclusion criteria, and created the research instruments (i.e., the information sheet), ensuring they were understandable to the target groups. The interview guide and codebook for the first data collection phase were also jointly formulated during this *preparation phase*.

Following this, the *data collection and analysis phases* commenced using the DEPICT approach (see section 2.7 and Table 1 in the Supplementary materials for further details). Accordingly, Sessions 5 and 6 focused on participatory data analysis. Between these sessions, consensual coding was conducted within a mini-team consisting of one academic and one non-academic member, who initially coded the data independently and subsequently reconciled their analyses. Session 7 served to compile the EI findings into a consensus document prepared by the academic team and refine the interview guide and codebook for the FGDs.

**Table 1:** Synthesis statements derived by the PAART research team

Synthesis statements	Collaboratively formulated statements	Source of data
Synthesis statement 1	The integrative analysis of all PAART data sources reveals the growing need for scientifically grounded, couple-oriented counseling.	Expert interviews Focus group discussions Systematic review
Synthesis statement 2	Both participant groups identified couples dealing with an initial MS diagnosis as the target group in particular need of support.	Expert interviews Focus group discussions
Synthesis statement 3	“Inhibiting factors” such as maladaptive disease processing, fear of emotional confrontation, and concerns of disclosing too much should be considered when planning counseling sessions.	Expert interviews Focus group discussions
Synthesis statement 4	Partnership strength was consistently identified as a key predictor of how couples living with MS cope with both initial diagnosis and disease progression.	Expert interviews Focus group discussions
Synthesis statement 5	Most participants from both groups identified communication as a crucial coping factor (especially during dynamic disease phases) and recommended actively practicing communication, particularly regarding difficult topics.	Expert interviews Focus group discussions
Synthesis statement 6	Both participant groups recommended that greater emphasis be placed on couple-related issues (rather than exclusively on medical aspects) when designing the content of counseling interventions.	Expert interviews Focus group discussions
Synthesis statement 7	The “uncertain future” is a central theme in couples’ experience of coping with illness and should be explicitly addressed in counseling formats.	Expert interviews Focus group discussions
Synthesis statement 8	Regarding the structure of interventions, couples expressed a strong desire for opportunities to exchange experiences, as well as a flexible format incorporating interactive, communication-enhancing exercises led by an authentic and empathetic facilitation team.	Expert interviews Focus group discussions
Synthesis statement 9	EI and FGD data revealed no clear preference regarding the timing (compact or extended) or delivery mode (virtually or in person) of counseling sessions.	Expert interviews Focus group discussions
Synthesis statement 10	The role of the facilitator—or facilitation team, which was preferred—was considered crucial in such counseling sessions; this was particularly emphasized by the couples.	Expert interviews Focus group discussions

EI = expert interview; FGD = focus group discussion

Following the FGDs, Sessions 8–10 focused on collaborative data analysis. Mini-teams conducted consensual base coding of the transcribed discussions, with each team coding one transcript. Each mini-team deliberately integrated an academic member, a practice-based member, and an experiential member of the PAART research team to strengthen triangulation and analytical rigor. Additional, separate meetings supported this process; analytical impressions were documented. After fine-coding (Session 9), the results were summarized in a consensus document drafted again by the academic team and discussed in Session 10.

Session 11 of the *synthesis phase* focused on critically evaluating the overall project. The team synthesized insights from all data collection phases, consolidated the key findings in a final document (synthesis statements), and developed collaborative strategies for translating and disseminating the results.

Across all phases of the participatory research process the PAART research team was continuously required to make decisions, for example regarding the formulation of interview questions, the coding of texts within the mini-teams, and the development of key findings and synthesis statements. Throughout these processes, particular attention was paid to ensuring that decision-making followed a democratic shared decision-making approach. Typically, this process involved an initial phase of idea generation, followed by a phase of deliberation and discussion, and culminated in a final democratic decision-making stage, often conducted through a show of hands and simple majority voting. When necessary, and where the project timeline allowed, decisions were occasionally postponed in order to allow for further reflection and discussion.

### 2.6 Data collection

#### 2.6.1 Context, participant engagement, and mode of data collection

Following nationwide recruitment efforts—conducted through newsletters and website postings of the German MS Society, social media, and flyers distributed at counseling centers—EIs were conducted with counselors from the German MS Society’s counseling service centers. These counselors were specialized in working with pwMS and their relatives. In the subsequent phase of data collection, FGDs were held with couples living with MS. For logistical reasons, all assessments were conducted remotely, using telephone interviews for the EIs and digital video conferencing for the FGDs. The EIs lasted an average of 33 min and 53 s (range: 28–41 min); the FGDs lasted between 79 and 89 min (mean duration: 84.75 min).

The EIs and FGDs were conducted by members of the academic research team. JN is a certified systemic therapist and counselor with extensive experience in patient-centered communication, and AKF has extensive experience in facilitating FGDs. The FGDs were conducted by two facilitators; the second facilitator was primarily responsible for notetaking and documentation.

With written consent obtained in each case, the EIs and FGDs were audio-digitally recorded and transcribed by an external transcription service (smoothed verbatim transcription). The counselors did not receive monetary compensation; however, the interviews were conducted during their regular working hours. The couples were compensated 60 EUR each.

#### 2.6.2 Participant inclusion criteria

Inclusion criteria for counselor participation in the EIs were as follows: a minimum of 1 year of professional counseling experience primarily working with clients living with MS at a German MS Society counseling center. Counselors who themselves lived with MS were also eligible to participate. Inclusion criteria for participation of couples living with MS in the FGDs were as follows: same- or different-sex couples in a close, personal (intimate) relationship for over 6 months who shared a household, with one partner having received a diagnosis of MS more than 6 months prior. Only pwMS with the capacity to provide informed consent were included. PwMS were excluded if experiencing a relapse or undergoing inpatient treatment at the time of data collection.

#### 2.6.3 Participant characterization

Information was collected for comprehensive characterization of the participants. For the counselors, the following data were gathered: demographic characteristics (age and sex); professional fields (employment scope in hours, counseling scope in percentage, and range of services); and qualifications (education and years of professional experience).

For the couples living with MS, the following data were obtained: demographic characteristics (age and sex of each partner); disease-specific data (year of initial MS diagnosis, MS course, three currently dominant symptoms, medication level: baseline or escalation therapy, disability scores: Expanded Disability Status Scale, (29), Patient-Determined Disease Steps (30), and level of care dependency); occupational data (job title, professional level, and employment status: capable of working >6, 3–6, or <3 h per day, retired); couple-related data (marital status, duration of partnership, duration of shared household, number of years living with the illness together, and children: number/age/living in the same household); and experience with (couples) counseling.

### 2.7 Data processing and analysis

The PAART study followed a predefined design: the study was conceptualized by the PAART research team, with the type and sequence of qualitative methods explicitly specified. An *a priori* defined sample size of 10 expert interviews (EIs) and four focus group discussions (FGDs)—each comprising four couples with varying ages and levels of disease severity—was initially considered sufficient to generate a comprehensive thematic corpus. In line with the approach proposed by Curry and Nunez-Smith (27), decisions regarding thematic sufficiency in relation to the research questions were made collaboratively by all members of the PAART research team following each phase of data analysis. In particular, the counselors and affected individuals within the team were involved in assessing thematic sufficiency and confirmed that the themes derived from the datasets adequately captured their professional practice and everyday lived experiences. Participant characteristics were analyzed using descriptive statistics. Transcribed expert interview (EI) and focus group discussion (FGD) data were analyzed using qualitative deductive–inductive content analysis (31, 32). An *a priori* codebook was developed by the PAART research team based on their counseling experience. Team members initially proposed interview questions, which were subsequently discussed and thematically clustered into overarching categories during a joint meeting. For the EIs, the categories included: (1) offers and invitations; (2) requests and expectations; (3) content; (4) organization and delivery formats; (5) objectives, target groups, and recruitment strategies; (6) features enhancing attractiveness; (7) agenda setting; and (8) design and structure. Based on this framework, the academic research team drafted the interview guide, which was reviewed and finalized collaboratively. The interview guide and codebook for the FGDs were subsequently refined based on insights from the EIs. Questions specific to counseling settings were removed, and additional questions were added regarding concerns about participation in couple-oriented counseling, expectations toward facilitators, and criteria for evaluating session benefits. The FGD categories comprised: (1) pathways and motives; (2) offers and invitations; (3) wishes, needs, and goals; (4) content; (5) organization and delivery formats; (6) objectives and target groups; (7) features enhancing attractiveness; (8) design and structure; and (9) perceived benefits.

The predefined codebooks were inductively expanded during data analysis within the participatory DEPICT framework (33). DEPICT comprises six sequential steps, ranging from dynamic reading and engaged codebook development to participatory coding, inclusive data review, collaborative interpretation, and translation. During basic coding, the container categories were differentiated into main themes and subthemes, while overarching themes reflecting dominant concepts across the dataset were identified during fine coding. The resulting thematic landscape reflected both the breadth of topics addressed and the frequency with which they were mentioned. Themes emerged through consensual coding within mini coding teams, direct category formation from the text, and focused summarization techniques, supported by MAXQDA® (34). Throughout the DEPICT process, analytical rigor and reflexivity were strengthened through shared decision-making within both the mini coding teams and the broader PAART research team. An overview of the six DEPICT steps, including guiding questions and tasks for the PAART teams, is provided in Supplementary Table 1.

### 2.8 Rigor and trustworthiness

The PAART study adhered to established quality standards for qualitative multistage research to ensure rigor and trustworthiness of data analysis (27, 35): (1) *Veracity* (credibility) was enhanced through triangulation at two levels: different participant groups (data source triangulation) and a multi-perspective research team involved throughout design development and data analysis (investigator triangulation). Additional strategies included participant confirmation of thematic sufficiency by the PAART research team and consensual participatory coding. (2) *Consistency* (dependability) was ensured through comprehensive documentation of the study context and processes, complemented by a *“*critical friend” approach—using an analyst from outside the research field to review adaptations and decisions throughout the study. (3) *Applicability* (transferability) was enhanced by detailed reporting of sampling procedures, participant characteristics, study settings, and data collection and analysis processes, enabling reader assessment of the relevance of findings to other contexts. (4) *Neutrality* (confirmability) was maintained by employing a PHR approach with a multidisciplinary team of individuals living with MS, healthcare professionals, and academic researchers.

## 3 Results

### 3.1 Participant characterization and recruitment: EIs

Semi-structured interviews were conducted between July and September 2022 with 10 counselors from German MS counseling centers (eight by telephone, one via video call, and one in person). Participants were recruited from six federal states and all were female; one counselor also lived with MS. Counselors had a mean age of 47.5 years (range 33–68) and an average of 10.8 years of counseling experience (range 1.3–30). Professional backgrounds included psychology, social work, nursing-related professions, occupational therapy, and related fields. Several participants had additional qualifications in systemic or person-centered counseling.

### 3.2 Key findings of the EIs

The following key finding reflects the consensus framework developed through investigator triangulation of the counselor expert interviews within the PAART research team and is supplemented by illustrative quotations:

(1) During the initial recruitment contact, all participating counselors expressed strong support for scholarly engagement with couples counseling and their desire to contribute through their participation.

*Quotation:*

*“What I think is really good is the topic of couples counseling in general, because there really is not that much focus on it due to the lack of time. That’s why I think it’s really good that they are taking it on and that it can be broken down further to the individual associations such as the [organization] or the regional associations so that we can also participate. That would be great!”*

(2) Counselors represented diverse professional and educational backgrounds, which was reflected in the broad range of topics identified and the nuanced problem descriptions they provided when asked about typical counseling issues faced by couples in their professional practice.
  *Quotations*:

*“A lot of it is also about what rights they have if they already have a care level assigned. I’m shocked every time by how uninformed people are. They get a care level, and that’s it. And somehow, they barely know about the funds and support they can actually claim.”*

*“Yes, absolutely. But I notice it very strongly in the area of mobility in particular. Also in the relatives’ group. People are very worried about what will happen if mobility becomes even more restricted. Especially with regard to participation. Including joint participation. The social circle will somehow become smaller and smaller. That’s a very common issue. And of course, even when the question comes up: How do I deal with this, when might the time be right to think about a care facility?”*

(3) Counselors report that outreach couples are particularly characterized by identifiable factors such as younger age, an early-stage or newly diagnosed illness (especially with fulminant onset and high initial care needs), higher levels of education, and greater openness in communication and engagement; additionally, single women after partnership are more likely to seek outreach support.

*Quotations*:

*“I have the impression that the young MS couples are more likely to be aware of this … they deal with the issues very differently anyway … they are much more open … with the older sufferers, it was more often the relatives who came together to form a group.”*

*“Of course, I have not checked it statistically, but I would say that two-thirds of the callers are women and more than half of them are single and often after a partnership, so to speak, i.e., that the illness has led to the end of the partnership.”*

(4) Couple-oriented themes, which refers primarily to interpersonal dynamics within couples were frequently highlighted, particularly boundary setting, experiences of overburden, shame, and concerns about the future. In contrast, disease-related themes directly associated with living with multiple sclerosis—including symptom burden, disease progression, treatment-related issues, and care needs—received comparatively less attention from the counselors.

*Quotation:*

*“(…) the husband had become ill, and the wife really enjoys mountain biking, riding up and down the mountains. And he always wants to come along. At that point, he was not yet severely affected. She says, ‘He drives me crazy. I’m constantly checking whether he can still manage.’ That takes all the enjoyment away for me. I need the joy of riding up there. And then he should maybe ride around below or do something else. I’m not angry or anything, but I don’t want to constantly watch whether he can still do it or whether I should perhaps stop after all.” … Yes, and I also think one could say that perhaps he was not even that interested in cycling in the first place, but with the illness in mind, there was this pressure to prove that he could still make it up there after all.”*

(5) Counselors reported that potential concerns regarding participation in such sessions—especially fears of emotional overwhelm—should be addressed early.

*Quotation:*

*“Yes. Well, I think there are also simply fears on the part of those affected that they will somehow be confronted too much with everything. That they will somehow have to do something. And that it might also overwhelm them emotionally to have to deal with the illness (…). I think that’s simply a very big challenge.”*

(6) Several Counselors reported that the quality of the premorbid relationship is a crucial factor in coping with the illness: MS was reported to present couples with coping challenges and to function as a “magnifying glass” for pre-existing dysfunctional relationship patterns.

*Quotation:*

*“But it can also be really independent of MS; it doesn’t necessarily have to be linked to MS, I’m going to say, but also when I notice that there is a dynamic that perhaps already exists long before the MS but we should also look at because it is simply also a burden.”*

(7) Communication within the partnership was repeatedly described as a key competence—or even a “guarantor” of relationship stability—particularly during the diagnostic phase and periods of relapse or disease progression. In addition, experiences of “speechlessness” or difficulties communicating were discussed in relation to shame-associated disease topics, such as incontinence.

*Quotations:*

*“(…) so I think the most important thing is communication … that couples learn to listen to each other again.”*

*“Well, I can remember a client who told me quite honestly that she simply hadn’t told her husband certain things yet, that it’s the way it is, for example in the area of incontinence or something.”*

*“And, of course this too: talk to each other, right? Have the courage. Say I’m fine or not fine, right? That’s always the case: “Oh, I’m doing great today; everything’s fine.” And, of course your partner realizes that it’s not, right? And that stirs up so many conflicts and fears, doesn’t it?”*

Please see Supplementary Table 2, which presents the inductively derived main themes and subthemes together with illustrative quotations from the complete interview material with counselors.

### 3.3 Participant characterization and recruitment: FGDs

Between June and September 2023, four FGDs were conducted via video conference with 16 couples (four groups of four couples). A total of 35 couples expressed interest, and participants were assigned consecutively to ensure heterogeneity regarding age and illness severity. The sample was drawn from five German federal states, with most participants from North Rhine-Westphalia. Among the 16 pwMS (13 female), the median age was 43 years (range 27–63), and the median disease duration was 10 years (range 0–21). Disability levels varied (mean EDSS 3.56, SD 2.23; mean PDDS 1.80, SD 1.62), and MS subtypes included relapsing–remitting (*n* = 10), secondary progressive (*n* = 4), primary progressive (*n* = 1), and unclassified MS (*n* = 1). Symptom burden was heterogeneous, spanning motor, sensory, autonomic, fatigue-related, cognitive, and speech/language domains. Regarding employment, seven pwMS were working full time, five part time, two were fully retired due to disability, two partially retired, one unemployed, and one on parental leave. Partners had a median age of 42.5 years (range 29–60). Twelve were employed full-time, two part-time, one retired, and one unemployed. All participants were in different-sex relationships, with an average relationship duration of 16.5 years (SD 9.8; range 1–34). Most couples were married (*n* = 11), six had children, and in the majority of cases (*n* = 14) MS was diagnosed during the relationship.

### 3.4 Key findings of the FGDs

The following key finding reflects the consensus framework developed through investigator triangulation of the FGDs within the PAART research team and is supplemented by illustrative quotations:

(1) All participating couples welcomed scholarly engagement with couples counseling and expressed support through their participation.

*Quotations*:

PwMS: *“I just wanted to say…the nice thing about this session was that I really felt perceived as a couple with the illness and I think that’s something that might be good in a seminar like this…for sure”*

Partner: *“What I take away from this is actually a good feeling that, firstly, people are engaging with the topic, and during the exchange I had the impression that we share similar thoughts and similar needs. I always find it reassuring to know that you are not alone.”*

(2) “Uncertainty about the future” emerged among couples as a central, unifying concern across all areas discussed. The unpredictability of the disease—particularly symptom exacerbation—along with the physical, psychological, social, and economic consequences of disease progression, poses significant challenges for pwMS and their partners.

*Quotations*:

PwMS: *“I think, especially at our age, there’s a lot of planning for the future, what’s coming up, children yes/no, can you manage it, cannot you manage it or something else, what could it look like in the future, if you are looking for an apartment, do you have to make sure you have an elevator or not. So somehow all of these issues keep you busy and I think it’s always important that relatives have their own space.”*

Partner: *“The interest is simply … that at the beginning you naturally have a lot of questions about how to deal with such an illness. Especially when you are young, you naturally have a few questions that do not just concern you for 10 years, but for 40 or 50 years, and you want to prepare yourself for that as long as you can.”*

(3) Considerably more relationship-oriented themes (e.g., the role of partners, navigating the balance between giving and accepting help, distinguishing between illness-related and relationship issues, managing expectations, and improving communication) than disease-specific issues were identified. However, lifestyle-related aspects, social legislation, and employment-related concerns were also considered relevant.

*Quotations:*

PwMS: *“Does it make sense to take out a 30-year loan, like some of our friends might do for an apartment or a house or something, when you do not even know if you’ll be able to pay it back?*

Partner: *“…many people find it difficult, especially with their partner, to talk about things such as the limitations they are experiencing, things that are happening, the help they need or wish for—or do not wish for. It is about opening up that space and gaining the sense of safety needed to begin these conversations with each other, so that silence does not gradually creep in.”*

(4) Most participants identified couples in which one partner was recently diagnosed with MS as the primary target group for counseling sessions; there were also arguments for including couples differentiated by age and life stage.

*Quotations:*

PwMS: *“I also think it’s good that people are even thinking about offering something for couples. That’s why we were quickly enthusiastic about it. I just hope that a format is developed from this quickly and that an offer is created for newly diagnosed individuals, so that they have an easier time dealing with the illness than we all did.”*

Partner: *“I think the first step is, of course, a recent diagnosis, so that they can get to grips with the subject, because the uncertainty is particularly great at the beginning. If you’ve already spent a few years together and have the diagnosis, then it’s already settled in a bit. Of course, there are always fears, uncertainties or other things or new topics such as wanting to have children or something like that, but especially at the beginning there is still this great unknown.”*

(5) While participants supported the idea of counseling sessions for couples living with MS, they also noted potential emotional barriers such as a tendency to avoid thinking about the future and the fear of gaining distressing knowledge.

*Quotations*:

PwMS: *“So my fear would be that the more informed my husband is about the situation, the more fears he might get that he does not have because he doesn’t have the knowledge.”*

PwMS: *“Yes. There is also a certain fear of … what one might see and what it could mean for oneself, especially when you are currently at a stage where you can still live relatively well with the condition. That is why, personally, I am not particularly eager to make use of such services (…) as long as I am not more severely affected.”*

(6) Peer exchange among couples was identified as a key element of the proposed counseling format, as participants emphasized the value of sharing experiences with others facing similar challenges within a supportive and empathetic environment.

*Quotations:*

PwMS: *“I’m ‘Team Weekend’, of course. I think it’s really great when you can still chat privately in the evening over dinner or something.”*

Partner: *“I think it’s great that we got the whole of Germany together and now three other couples that we didn’t know before have come out of the woodwork a bit and that we could simply… exchange ideas.”*

(7) Participants expressed strong openness to interactive approaches and emphasized the need for flexibility in thematic content and group composition. A good balance between problem-oriented and resource-oriented approaches was considered particularly important.

*Quotations:*

PwMS: *“I would also appreciate it if it were a bit more resource-oriented—so not just having a room and saying, “Now we’re doing a seminar, now we are doing counseling, and now we’re putting all the problems on the table”—but really looking at what strengths already exist in the relationship.”*

Partner: *“PowerPoint usually makes the first impression on me. It’s a bit like an info flyer: for a first summary basic knowledge, okay, and when it goes further, it’s not as conclusively helpful as personal interaction.”*

(8) With regard to the organization of the program, participants considered optimal group sizes to be four to five couples for virtual sessions and five to ten couples for in-person meetings. There was also considerable support for intensive weekend sessions as well as recurring monthly sessions scheduled on weekday evenings (e.g., 5–7 p.m.).

*Quotations:*

PwMS: *“So I would actually be in favor of once a month, because you might want to bring something with you until the next meeting, perhaps in terms of topics or a bit of observation of what the others have said.”*

Partner: *“(…) a no-go is when it’s such a huge group and you somehow never get a chance to speak because 16 people are sitting there.”*

Please see Supplementary Table 3, which presents the inductively derived main themes and subthemes alongside illustrative quotations from the complete FGD interview material.

### 3.5 Synthesis

Following evaluation of the findings from both data collection phases (EIs, FGDs), the PAART research team derived the following synthesis statements (1–10). At this point, findings from the concurrently conducted systematic review (18) were also incorporated, the PAART research team derived the following synthesis statements (1-10). At this point, findings from the concurrently conducted systematic review (18) were also incorporated (see Table 1).

## 4 Discussion

The PAART project aimed to explore requirements, expectations, and perceived attractiveness factors of couple-oriented counseling services through expert interviews and focus group discussions involving key stakeholder groups, namely 10 experienced counselors and 16 couples living with MS. The study was guided by a participatory health research (PHR) approach, actively involving representatives of the target groups as co-researchers in the data analysis process. To our knowledge, this represents the first qualitatively driven participatory study specifically examining the development of outpatient couple-oriented counseling services for couples affected by MS. The synthesis of the PAART findings generated the following main implications for the design and implementation of outpatient counseling interventions for this population.

### 4.1 Implications for the design and implementation of outpatient counseling interventions

#### 4.1.1 Implications for early couple-oriented counseling and participation barriers

There is a substantial unmet need for scientifically grounded, couple-oriented counseling for people affected by MS, as emphasized by both counselors and couples. In addition, our systematic review revealed that compared with other chronic conditions—most notably cancer (36, 37)—scientific understanding of the relevance and effectiveness of couple-oriented counseling in the context of MS remains limited due to the paucity of intervention studies (18) *(synthesis statement 1)*.

Both participant groups identified couples dealing with an initial MS diagnosis as the group in particular need of support *(synthesis statement 2)*. Couples characterized this phase as an “emotional state of emergency,” a “moment of shock,” and a “loss of control,” accompanied by considerable uncertainty and numerous questions on both sides. Participants emphasized that sensitive and individualized MS-related education may help reduce fears during this phase. In line with this, Wawrziczny et al. (38) highlighted the importance of providing support early, before avoidance patterns become entrenched. Esmail et al. (39) described “emotional buffering,” whereby partners attempt to protect one another from emotional distress, potentially limiting open communication and preventing the development of shared coping strategies.

At the same time, our data identified several barriers to participation in couple-focused counseling, including fear of emotional confrontation and concerns about disclosing illness-related information *(synthesis statement 3)*. Some couples expressed discomfort regarding how much disease-related information should be shared with their partner. Such concerns may hinder coping and future planning processes, as Rolland (40) found. Participants therefore emphasized the importance of addressing these concerns early in the counseling process and establishing emotionally safe communication structures within the group setting.

#### 4.1.2 Implications for dyadic coping and navigating uncertainty

The PAART findings underline that the quality of the partnership is a central determinant of how couples cope with MS *(synthesis statement 4)*. This is consistent with empirical evidence (41, 42) and established dyadic coping models (43–45). Together, these approaches emphasize that coping with chronic illness should be understood from a dyadic perspective (46, 47). Given the unpredictable and progressive nature of MS, couples living with MS in particular are repeatedly required to renegotiate roles, needs, and coping strategies. This results in an ongoing process of relational adjustment and requires continuous rebalancing between stability and adaptation (48, 49). Couple-oriented counseling should therefore support partners in understanding these dynamics and strengthening shared coping processes. Boland et al. (50) noted that healthcare professionals may also be insufficiently prepared to address the emotional complexities that arise within dyadic relationships. Similarly, Lewis et al. (51) found that couples affected by MS often preferred relationship-centered coping interventions that emphasized the strengths and benefits of the relationship rather than focusing primarily on coping with the disease itself. This perspective was also reflected in our data, as both participant groups recommended placing greater emphasis on couple-related rather than exclusively medical issues when designing counseling interventions *(synthesis statement 6)*. Counselors highlighted themes such as feeling “seen” within the relationship, conflict management, navigating daily life, role clarification, and experiences of shame and self-worth. Couples emphasized balancing support, distinguishing between illness-related and relationship concerns, and planning for the future together. Medical topics (e.g., medication use, immobility) played a comparatively minor role. Instead, participants focused on less visible MS-related symptoms, such as fatigue, cognitive changes, and incontinence, which often remain insufficiently addressed within intimate relationships. As these impairments become visible primarily through communication, participants underscored their strong social and relational dimension.

“Navigating disease-related uncertainty together” emerged as a key overarching theme of couple-oriented counseling *(synthesis statement 7)*. Consistent with previous research on individual fear of progression in MS (52–54), participants described disease unpredictability and concerns about future physical, psychological, social, and financial consequences as major stressors for both partners. Timely information, mutual exchange, and joint future planning were identified as important coping strategies, particularly by the focus group participants. Counseling interventions should therefore explicitly address future-related concerns, including financial and housing issues, changing roles, and dyadic coping processes, while supporting couples in developing shared strategies for managing uncertainty over time.

#### 4.1.3 Implications for the design and organization of a couple-oriented counseling program

The PAART study data indicate that couples prefer flexible, interactive, and practice-oriented intervention formats that emphasize experiential learning, peer exchange, and communication-enhancing exercises *(synthesis statement 8)*. One counselor highlighted the importance of fostering open communication within couples, stating: “Talk to each other…have the courage!” This statement underscores the need for interventions to provide a safe and supportive environment in which couples can practice discussing sensitive topics, including areas where communication may previously have broken down, such as incontinence *(synthesis statement 5)*. These findings are consistent with the review by Busch et al. (13), which emphasized that interventions designed to support dyadic coping in the context of MS should strengthen both partners’ emotion-focused and problem-focused communication skills. In line with this, an exercise-based seminar format centered on interpersonal interaction may be more effective than purely educational approaches, as also suggested by Hartman et al. (55) in the context of chronic illness.

Participants further emphasized that such programs should be delivered by an authentic, empathetic, and professionally competent facilitation team. Notably, the definition and positioning of facilitator roles emerged as a highly salient theme during the focus groups *(synthesis statement 10)*. Participants expressed a clear preference for facilitators with strong MS-specific expertise who are capable of effectively guiding discussions while fostering empathy, structure, and emotional safety within the group setting. In particular, a dedicated facilitation team with a clearly defined separation from research-related roles was considered essential for creating trust and openness among participants. This underscores the importance of role separation and deploying a team whose respective responsibilities are clearly communicated to participating couples beforehand. With regard to program structure, no clear preference emerged between compact versus serial formats or between virtual and in-person delivery modes *(synthesis statement 9)*. Both intensive weekend-based interventions and recurring evening sessions (e.g., 5–7 p.m.) were perceived as feasible and acceptable. Suggested group sizes ranged from four to five couples in virtual settings and five to ten couples in face-to-face formats. While participants acknowledged the value of predefined core topics, they also emphasized the importance of maintaining sufficient flexibility to adapt content to the specific needs and dynamics of each group.

### 4.2 Strengths and limitations

#### 4.2.1 Methodological contributions and rigor

The participatory health research (PHR) approach constituted a key methodological strength of this study. Representatives of the target groups were actively involved as co-researchers throughout the development of the study design, data collection, and the analysis, interpretation, and synthesis of findings. This collaborative process strengthened the practical relevance and contextual validity of the results, as the stakeholder contributed insights grounded in both professional expertise and lived experience, thereby enabling informed interpretations beyond established facts (56). The participatory process was experienced as highly collaborative and co-creative, as reflected in the sustained engagement and absence of dropouts among co-researchers despite the long duration of the study. Furthermore, the study design integrated counseling expert interviews and focus group discussions with people affected by MS in order to capture the complex life worlds of couples living with the disease. This methodological approach enabled an in-depth exploration of lived experiences, support needs, and diverse perspectives on couple-oriented counseling and support services. The findings extend previous research highlighting that couples’ adaptation to chronic illness should be understood within a broader health and social care context. Wright et al. (49) described this broader perspective as a “second-degree system perspective,” in contrast to a “first-degree system perspective” limited to processes occurring solely within the couple system. Accordingly, each couple’s adaptation to MS should be conceptualized within a systemic–ecological framework that includes social and professional support structures, thereby facilitating more effective rehabilitation and adjustment processes (57).

#### 4.2.2 Limitations: sample characteristics and transferability of findings

The study involved highly motivated couples, as reflected in the particularly open and in-depth discussions during the focus groups. However, these couples may differ from those experiencing greater relational strain or more severe illness-related burden, which may limit the transferability of the findings. Moreover, most participating pwMS reported mild to moderate impairment and remained employed; consequently, the experiences of couples affected by more advanced disease stages may be underrepresented. Thus, care-related topics, such as caregiving burden, coordination of daily care routines, dependence on formal care services, may gain greater relevance in a sample including more severely affected individuals. Furthermore, 13 of the 16 participants affected by MS were female PWMS. Future research may therefore benefit from subgroup-specific focus groups (e.g., individuals whose relationships ended following the onset of MS, couples in which one partner has substantial MS-related care needs, or male PWMS) in order to explore context-specific experiences in greater depth, as it may be assumed that distinct themes relevant to couple-oriented counseling programs emerge within each subgroup. In addition, non-cohabiting, same-sex, binational, and culturally diverse couples were not represented in the sample, thereby restricting the diversity of perspectives captured in the dataset. The study further lacked access to objective clinical data on disease severity, which could have enabled a more precise clinical characterization of the sample.

The PAART study included highly motivated and reflective counselors with diverse professional backgrounds, thereby providing a broad range of perspectives and insights. However, all participating counselors were female, which may have introduced a gender-related bias toward couple-relevant topics and perspectives. Moreover, the counselors worked within a specific professional association structure embedded in the German healthcare system, which may limit the transferability of the findings to other healthcare settings.

#### 4.2.3 Challenges of participatory research processes

The PAART project also highlighted several practical challenges associated with participatory qualitative research. Although co-researchers were actively involved in data interpretation and synthesis, their limited prior experience with qualitative analysis required substantial preparation, methodological guidance, and structured moderation by the academic research team. As a result, levels of participation varied across different phases of the study. According to the participation framework proposed by Wright et al. (24), participation during data interpretation and synthesis may be characterized as co-learning and co-decision-making, whereas parts of the coding process remained closer to a consultation level due to the need for more intensive methodological support. Such an empowerment process may enhance interrater reliability; however, of greater importance are the democratically balanced decision-making processes among members of the multiprofessional research team. To reduce researcher bias, it would—in retrospect—have been helpful to appoint, on a rotating basis, a member of the co-researcher team to observe this “democratic process” from an external perspective and intervene whenever the researchers exerted too much influence.

Finally, the study demonstrated that participatory research processes require considerable organizational resources. Meaningful and equitable participation depends on accessible methodological support, transparent communication structures, shared access to relevant information, and effective facilitation to ensure that diverse perspectives can be integrated into consensus-oriented decision-making processes. Future long-term participatory studies involving multiple stakeholders should allocate sufficient time and resources for training, coordination, and collaborative reflection throughout the research process to facilitate meaningful participation.

## 5 Conclusion

This study highlights the substantial need for couple-oriented counseling services for people affected by MS. Central themes identified across participant groups included navigating disease-related uncertainty, strengthening dyadic coping, and promoting open communication within the relationship. The findings further emphasize that counseling interventions should be flexible, practice-oriented, and tailored to the specific needs of couples across different stages of the disease trajectory.

The qualitative participatory design enabled an in-depth exploration of couples’ and counselors’ perspectives and provides an important foundation for the development of evidence-informed counseling interventions for couples living with MS. Future research should evaluate the feasibility, acceptability, and effectiveness of such interventions across different healthcare settings and more diverse couple populations.

## Data Availability

The raw data supporting the conclusions of this article will be made available by the authors, without undue reservation.
