# Supplementary material for: Navigating uncertainty together: a participatory mixed-method study of counseling services for couples living with multiple sclerosis
Source: Front Public Health. 2026 Jun 24;14:1816630. doi: 10.3389/fpubh.2026.1816630 (PMC13343353; doi:10.3389/fpubh.2026.1816630)
Supplement: Supplementary file 1 [file Data_Sheet_1.PDF]

Table 1. DEPICT steps, guiding questions, and to-do tasks for PAART teams

DEPICT step	Question to ask	To-do's: Academic team	To-do's: PAART research team	To-do's: Mini-team
D-ynamic reading<br><br>(before session 5 and session 8)	- What are our initial impressions after reading the data?<br>- Which contents are most relevant to the main research question?	- The academic team assigns each member of the PAART research team three EI transcripts (stage 1) or one FGD transcript (stage 2) for preliminary review	- Each individual member receives three EI transcripts (stage 1) or one FGD transcript (stage 2) for preliminary review	-
E-ngaged codebook development<br><br>(during session 5 and during session 8)	- Are our categories appropriate or do they require revision or expansion?<br>- Do we have a shared understanding of each category's meaning and application?		- Development of the codebook and clarification of categories within the research team,<br>- Discussion of the coding procedure	-
P-articipatory coding<br><br>(between sessions 5–6 and 8–9)	- Which transcript sections align with each category in our category tree?	- Assignment of coding teams:<br>EIs: one academic researcher and one co-researcher<br>FGDs**:** one academic researcher and two co-researchers (one pwMS/partner and one healthcare professional)	-	- In mini-teams, each researcher initially coded the data independently, then compared and aligned categorizations
I-nclusive reviewing and summarizing of categories	- What are the main ideas?<br>- Where are coding inconsistencies?<br>- What are the key quotations?		- Presentation and collaborative review of initial coding, with clarification of any issues or difficulties<br>- Collaborative evaluation of categories and identification of	-

(during session 6 and during session 9)	- Are there notable silences or absences that should be noted?		meaningful subcategories (“fine coding”)	
C-ollaborative analyzing<br><br>(during session 7 and during session 10)	- What does this mean?<br>- What are the key findings?<br>- What questions remain?	- The academic team summarized the key findings from the EIs and the FGDs, each into a separate consensus document	- Discussion of consensus paper(s) and clarification of possible additions<br>- Stage 1 only: Identification of areas where the FGD guide needs revision or expansion	-
T-ranslating<br><br>(during session 11)	- Who are the target audiences, and how can we reach them?<br>- Who are the most effective messengers?<br><br>-		- The team synthesized insights from all data collection phases and the parallel systematic review in a final document (synthesis statements)<br>- Development of a translation and dissemination plan for sharing research results with relevant stakeholders (e.g., selecting journals and conferences, organizing training seminars for counselors, preparing informational brochures for people/couples affected by MS)	-

Note: pwMS = persons with multiple sclerosis; EI = expert interview; FGD = focus group discussion
