# Supplementary material for: Navigating uncertainty together: a participatory mixed-method study of counseling services for couples living with multiple sclerosis
Source: Front Public Health. 2026 Jun 24;14:1816630. doi: 10.3389/fpubh.2026.1816630 (PMC13343353; doi:10.3389/fpubh.2026.1816630)
Supplement: Supplementary file 2 [file Data_Sheet_2.PDF]

Table 2. Expert interview guide: categories, questions, example quotes, and inductive themes

Interview guide questions	Example quotations	Main themes/subthemes
	**Category 1: Offers and Invitations**	
**Do you generally offer couples a joint counseling service?**	C: Most of the time, we invite the respective partner to join us in the first or second session ... for example, when it comes to the topic of self-employment ... or differences ... "my partner doesn't understand me" or "patronizes me" ... or the couple is something for our "Plan B project" ... this is for couples who want to have children, and we make them aware of this very explicitly.	***Diverse formats of couple-oriented counseling services***<br>(a) Direct offers for individual couples counseling<br>(b) Indirect offers resulting from individual counseling sessions with persons living with MS or partners<br>(c) Fixed programmatic couple seminar offers (e.g., "Plan Baby for MS")
**In your experience, what factors justify a couple-oriented counseling approach?**	C: In any case, everyday life is an issue. And a typical example that I have often come across is that an MS sufferer—usually a male sufferer—doesn't care about the disease at all. However, the partner is very worried and wants to help, but meets with resistance.<br><br>C: (…) But it can also be really independent of MS; it doesn't necessarily have to be linked to MS, I'm going to say, but also when I notice that there is a dynamic that perhaps already exists long before the MS but we should also look at because it is simply also a burden.	***Key indicators for recommending couple-oriented counseling***<br>(a) Primarily, discrepancies in perception of the illness/resolution and realization of the problem<br>(b) Signs of excessive demands (demarcation/change of role)<br>(c) (Pre-existing) conflicts and communication disorders<br>(d) Mastering "normal life"
**In what instances would you recommend psychological couples therapy?**	C: Yes, I also advised it. And I have to say, now during corona it's become extreme anyway, right? I'm just saying somehow, nine out of ten counseling sessions I really have to give a recommendation (...)<br><br>C: (…) But, of course I can't take someone straight to psychotherapy. If I could, I think the line would be burning up, right? I really have to say. I can only name a few places to go, where you can somehow get in touch, and of course explain the procedure, right? Many people don't know that either.	***Referral pathways for psychological couple therapy***<br><br>Cases of severe chronic conflicts are clearly indicated; highly contentious couples (and families) are also recommended.<br><br>In most cases, referrals are first made to German MS Society internal or cooperating psychologists or to external family counseling centers. Direct referrals to private psychotherapy practices are less common.

Category 2: Requests and Orders		
**Is counseling requested or desired by couples in your experience?**	C: (…) Or those who want to find out more, or where one person says, "I want to be prepared in any case and prepare myself," and the person affected perhaps even says, "First of all, I don't want to have anything to do with it yet."<br><br>C: (…) Especially when men are diagnosed, it's more the women who call when they are first diagnosed and say, "Here, my husband." (...).	***Gender-related driving forces behind seeking couples counseling***<br>There is always a "driving force" behind the desire for couples counseling, with women often the initiators—both in their role as the person with MS and in their role as a partner; this gender effect is not observed in younger people.
**What couples living with MS seek such counseling? Are there any identifiable demographic, disease-specific, or couple-related characteristics?**	C: I have the impression that the young MS couples are more likely to be aware of this ... they deal with the issues very differently anyway ... they are much more open ... with the older sufferers, it was more often the relatives who came together to form a group.<br><br>C: (…) Of course, I haven't checked it statistically, but I would say that two-thirds of the callers are women and more than half of them are single and often after a partnership, so to speak, i.e., that the illness has led to the end of the partnership.	***Identifying outreach couples: Youth, early-stage illness, education, and openness***<br>(a) "Outreach couples" tend to be<br>(b) younger couples<br>(c) couples with a new diagnosis (especially with a fulminant onset and high initial level of care),<br>(d) couples who are more highly educated,<br>(e) women who are single post partnership.<br>(f) Outreach couples are also characterized as "open couples."
**What are the motivations behind this? What "concerns/requests" and stated goals do couples bring to your consultations?**	C: I think those who are newly ill have a great need in any case. When the illness is still new, they need to find a way of dealing with it and coming to terms with it together. That's not just the job of the person with the illness or the people with the illness.<br><br>C: I've had a young lesbian couple, for example, where one of the women has now been diagnosed and came to me for counseling because she wanted a safeguard for her wife, so that the wife could come to us in a crisis. So things like that also come up here.<br><br>C: Sensing a connection between stress levels and her physical condition and disease activity. (...)	***Illness dynamics as a catalyst for counseling***<br>The "dynamics" of the illness (new diagnosis, relapses, progression) are often a motive for seeking counseling together.<br>(a) Further counseling assignments concern<br>(b) dealing with the new diagnosis,<br>(c) the topic of "changes" as well as future-oriented topics (security, the desire to have children),<br>(d) emotional regulation (anxiety/progression anxiety),<br>(e) coping with everyday life and stress,<br>(f) questions about sociomedical benefits, and care and provision of aids.

**What expectations and fears do couples share?**	C: Yes. Well, I think there are also simply fears on the part of those affected that they will somehow be confronted too much with everything. That they will somehow have to do something. And that it might also overwhelm them emotionally to have to deal with the illness (…). I think that's simply a very big challenge.	***Barriers to couples counseling: Emotional and cognitive inhibitors***<br>(a) "Inhibiting factors" in seeking couples counseling are observed<br>(b) in the context of maladaptive disease processing (e.g., denial, avoidance),<br>(c) where there are fears of emotional confrontation, and<br>(d) where the partner without the diagnosis feels that they are not entitled to counseling.
	**Category 3: Content**	
**What specific disease-related issues do couples living with MS bring to consultations?**	C: (...) for example, the issue of tiredness ... so I always go to bed first, sometimes I'd like to go to bed before my daughter ... and my husband is often down here in the living room all alone because I'm already asleep ... so that's definitely an issue for us here. (Counselor with MS).	***Disease-related topics***<br>(a) Dealing with a new diagnosis<br>(b) Managing disease progression<br>(c) Management of non-visible symptoms (e.g., fatigue, cognition/memory)<br>(d) Session-specific topics (e.g., "Family planning and children," including pregnancy and medication considerations)
**What specific couple-related issues do couples living with MS bring to consultations?**	C: (...) I mean, the person is more than the "MS" ... And yes, MS is often the hook here, of course, and then it often moves away from that and onto other topics that don't originally have anything to do with MS.<br><br>C: (…) So shame is a huge issue and that of course also makes communication an issue.<br><br>C: And one issue, I think, is also fear, i.e., my own fear of whether it will stay that way, I mean, everything has gone back to normal for me, but the fear that it could stay that way, and also for my partner, the fear that restrictions could remain now.	***Couple-related topics***<br>(a) Coping with the disease together—whether in-sync or out-of-sync—and including the experience of "being seen" both within the relationship and as individuals, with or without MS<br>(b) Partnership quality, communication, and dealing with conflicts<br>(c) Managing daily life and regulating stress (often referred to as "couple energy")<br>(d) Clarifying roles, boundaries, and addressing caregiver strain or burnout

	C: The different needs of those affected and their relatives are simply in the room. And that, for example, the partner is also worried that the person affected is not taking good care of themselves with their illness. (…)	(e) Emotional regulation, particularly regarding fear, shame, and grief—especially when mobility is lost<br>(f) "Couple identity and intimacy"<br>(g) "Creating a joint path to/shared vision of the future"
**In your experience, what other topics are relevant for couples?**	C: Yes, absolutely. But I notice it very strongly in the area of mobility in particular. Also in the relatives' group. People are very worried about what will happen if mobility becomes even more restricted. Especially with regard to participation. Including joint participation. The social circle will somehow become smaller and smaller. That's a very common issue. And of course, even when the question comes up: How do I deal with this, when might the time be right to think about a care facility?	***Other topics***<br>(a) Challenges at work/in the workplace<br>(b) Reduced earning capacity and pension-related issues<br>(c) Social law matters (including caregiving)<br>(d) Financial concerns<br>(e) Home adaptation<br>(f) Provision of assistive devices<br>(g) Nutrition and relaxation<br>(h) Self-care and resilience<br>(i) Barrier-free travel (with family/friends)<br>(j) Rehabilitation and therapy options<br>(k) Building support networks
**Category 4: Organization and Delivery Formats**		
**Based on your experience, which organizational format(s) do you find suitable for specific target groups of couples living with MS?**	C: We are trying it out ourselves and haven't found a good way yet because we assume that it's a family workshop. We also offered this for a weekend in March, where the children are looked after by my colleague or myself alone. We invited a speaker to talk about relaxation and interesting topics. We did a lot of canvassing to get 10 or 12 couples together. That was very, very difficult.<br><br>C: (...) We also have a seminar program. I used to run a camp for severely affected people, and once we took this couple with us so that they could have a break from everyday life. It's often just small things that you have to bring in to make things more positive again.	***Clarifying the target group and focus is important***<br>It is essential to define whether the offering is for newly diagnosed individuals or those with advanced care needs—and whether the emphasis is on shared experience or specific MS-related topics.<br><br>***Trial and error in reaching couples***<br>Many counselors are still experimenting with ways to engage couples, particularly in dyadic formats, especially as many face-to-face services were discontinued during the COVID-19 pandemic.
**What time-related organizational formats**	C: (…) But actually, what I often hear is that the evening appointments are not ideal. So, if you're planning a group like	***Timing matters: Avoid evening sessions***

**are feasible for couples based on your experience?**	this, you're often already tired and so on. Then I would perhaps go for Saturday mornings or weekday mornings rather than evenings.<br><br>C: Yes, exactly. The problem with one day is that it's too stressful during the week. They really put it off. We've already done surveys and it's very, very difficult. What is really good, thanks to me, are webinars. I really have to say, that's good. They don't have to leave their home environment. That's not necessarily great, because they need to get out into other situations to discuss things with others. But because everyone has time problems, it seems to be something suitable.	Evening formats are often experienced as rather unfavorable due to MS-related fatigue, cognitive challenges, and common scheduling conflicts such as work and childcare.<br><br>***Consistency and connection are key***<br>Allowing space for couples to share their stories was emphasized as important. It also helps to justify the potentially long trips required for attendance. Regular meetings should be offered.
**In your experience, what delivery format(s) do you find that couples living with MS prefer?**	C: I think it's good anyway if you have something like a mixture of just talking and practicing and also doing something and visualizing it, especially with groups.<br><br>C: I know that [person] was very enthusiastic about the weekend. Or maybe she's done it several times before. But they were in the hotel. Of course, it was also good for people to say, "Let's get out now; we don't have to worry about anything or anything."	***Engaging formats with practical considerations***<br>Positive experiences are associated with a diverse and engaging format, including inviting interesting keynote speakers, incorporating interactive elements (such as exercises, but avoiding role play), and using accessible venues or hotels with full catering.<br><br>However, the budgeting and cost coverage must be clarified in advance, and session spaces should be barrier-free.
	**Category 5: Objectives, Target Groups, and Advertising**	
**Why should couples attend counseling sessions?**	C: I think this time away from everyday life is particularly important. I would offer people a weekend seminar and also justify it by saying that a break is also good for once, in which you can perhaps rediscover yourself as a couple. I could imagine that being very effective, very deepening for the relationship if it's not in a crisis right now, but simply as prevention.<br><br>C: I think a counseling service like that is a good thing to help couples stay together, because it supports them, shows them	***Preventive and inclusive goals of counseling sessions***<br>The counselors identified several reasons for offering dyadic counseling sessions, including<br>(a) a fundamental lack of existing dyadic services,<br>(b) the preventive potential of such interventions, and<br>(c) the opportunity to reach and involve partners with MS.

	how to deal with it, takes them through phases of grief and explains it; that helps a lot.	
**Should couples be separated by age or disease severity?**	C: I wouldn't limit that to a specific group, I don't think. I think those who are newly ill definitely have a great need. When the illness is still new, they need to find a way of dealing with it and coming to terms with it together.<br><br>C: I find that difficult. I would separate the newly ill people a little. Because in my experience, meeting someone who is very seriously affected can trigger additional anxiety that also needs to be processed. So perhaps I would keep the newly ill people separate.	***Stage-specific challenges and needs***<br>There was broad agreement on the importance of distinguishing between different stages/phases of the disease—particularly between newly diagnosed individuals and those requiring a higher level of care—as these present distinct challenges, coping requirements, and everyday realities.
**In your opinion, how can couples living with MS be won over to outpatient counseling services?**	C: I might write down questions or thoughts that I assume the couple is experiencing. For example, "Yes, if you decide to go out, are you afraid that your partner's limitations will remain forever?" or "Do you sit alone in the living room in the evening because your partner is already in bed?" Or more generally, "Things haven't been the same since the diagnosis."<br><br>C: I think it's always important to look at how couples really come to counseling. After all, there is usually one partner who provides the impetus and wants to drag the other along. It's rarely the case that it's balanced. That you create a relaxed introduction, a relaxed invitation. I think that's the most important thing. A bridge to both.	***Effective communication and promotional strategies***<br>The German MS Society website and member magazine, flyers and posters (e.g., in doctors' offices), and social media platforms (e.g., Instagram, Facebook) are considered typical channels for promotion.<br><br>An appealing title (e.g., "Couple Time") and emotionally resonant phrases (e.g., "And suddenly, MS was in our lives" or "Things haven't been the same since the diagnosis") are recommended as effective presentation hooks.
	**Category 6: Attractiveness Features**	
**What "attractiveness features" would induce couples to participate in such sessions?**	C: I would offer people a weekend seminar and also justify it by saying that a break is also good for once, in which you can perhaps rediscover yourself as a couple. I could imagine that being very effective, very deepening for the relationship.<br><br>C: Then the question is, does it cost something or does it cost nothing? If it costs nothing, then you can communicate that.	***Incentives for participation***<br>The opportunity to "give the couple some couple time" to consciously invest in their relationship ("rediscovering the couple")—ideally "free of charge" in a barrier-free "hotel environment."

**Do you have any experience with digital counseling services? In your experience, would this be attractive to couples living with MS?**	C: We have only had positive experiences with digital formats here, because then the people concerned don't have to organize their attendance.<br><br>C: I'm not a fan of digital offerings in this area. But that's also very individual. Younger people in particular—okay, I'm still one of them—are a bit more open and find it quite good. But I find it more impersonal than meeting in person.<br><br>C: Yes, you can also withdraw from time to time. Some people meet up during the breaks and exchange ideas about what you can bring to the group. You get to know that when you are present.	***Digital formats are rated heterogeneously***<br>Digital formats have become more attractive following the COVID-19 pandemic, with face-to-face formats less preferred.<br><br>Digital formats save time and travel and are consistently more popular with younger people.<br><br>Nevertheless, digital formats are generally not in high demand as they are impersonal, do not allow the development of interpersonal dynamics (especially during exercises), and offer no space for informal exchange.
	**Category 7: Agenda Setting**	
**What disease-related topics would you cover during such preventive counseling sessions (regardless of format)?**	C: (...) the whole neuropsychological area, memory, concentration, in other words everything that you can't see, but which is of great importance in everyday life and especially in communicative relationships.<br><br>C: It is sometimes really difficult for the healthy person in the partnership to understand why someone sometimes lies on the couch all day.	***Disease-related topic setting***<br>Disease-related topics can be grouped into the following clusters:<br>(a) Coping with a new diagnosis and disease progression<br>(b) Managing invisible symptoms such as fatigue and cognitive challenges<br>(c) The specific topic of family planning, including pregnancy and medication
**What couple-related topics would you cover during such preventive counseling sessions (regardless of format)?**	C: (...) so I think the most important thing is communication ... that couples learn to listen to each other again. (...)<br><br>C: Well, I can remember a client who told me quite honestly that she simply hadn't told her husband certain things yet, that it's the way it is, for example in the area of incontinence or something.<br><br>C: And, of course this too: talk to each other, right? Have the courage. Say I'm fine or not fine, right? That's always the case: "Oh, I'm doing great today; everything's fine." And, of	***Couple-related topic setting***<br>Couple-related topics can be grouped into the following clusters:<br>(a) Coping with the illness together (synchronous/asynchronous)<br>(b) Communication and conflict<br>(c) Stress regulation, boundaries, and excessive demands<br>(d) Emotional regulation—including fear, shame, and grief<br>(e) Couple identity and intimacy

	course your partner realizes that it's not, right? And that stirs up so many conflicts and fears, doesn't it?	
**What, other than disease- and couple-related topics, would you cover during such preventive counseling sessions?**	C: (…) A lot of it is also about what rights they have if they already have a care level assigned. I'm shocked every time by how uninformed people are. They get a care level, and that's it. And somehow, they barely know about the funds and support they can actually claim.	***Other topics***<br>Other topics include financial issues, work, family, friends and travel, and social issues.
	**Category 8: Design and Structure**	
**Based on your experience, how should such sessions be structured in terms of content?**	C: If the couples are not currently in an acute crisis, but if it is intended more as a preventative measure, then a weekend event with couple exercises can be a really great new couple experience.<br><br>C: I think it's good anyway if you have something like a mixture of talking and practicing and also doing something and presenting it visually, especially in groups.	***Designing with flexibility and sensitivity: Diverse formats and topics***<br>A diverse session design is recommended,<br>(a) incorporating a range of topics (including external speakers),<br>(b) with varied settings (both in-person and online), and<br>(c) with flexible interaction formats such as introductions, paired exercises, visual representations, and group discussions.
**In your experience, what time and organizational formats are feasible for couples?**	C: (...) so I think Saturday mornings would be better, because fatigue hits hard in the evenings during the week ... and the couples might still need a babysitter (...)	***Balancing engagement and practical constraints***<br>Weekend sessions, offering couples a "time-out" together, tend to be more appealing.<br><br>When planning these sessions, important considerations include illness-related factors (e.g., fatigue, cognitive challenges) and everyday demands such as work, childcare, and day-to-day stress.
	C: (...) yes, I wouldn't take more than 4 or 5 pairs in such a group. (...)<br><br>C: That's why I think you can't do it as a complete layperson or something, right? It's good if you have a team, a	***Small groups, safe spaces***<br>Group sizes of 5 to 7 couples are considered ideal for fostering a trusting environment and enabling meaningful exchange within group dynamics.

	psychologist, social worker, doctor, whatever. The more multi-professional, the better, right?	Facilitators should have a therapeutic background; having a multi-professional team is viewed as an advantage.

Note: C = counselor; MS = multiple sclerosis.
