# Supplementary material for: Navigating uncertainty together: a participatory mixed-method study of counseling services for couples living with multiple sclerosis
Source: Front Public Health. 2026 Jun 24;14:1816630. doi: 10.3389/fpubh.2026.1816630 (PMC13343353; doi:10.3389/fpubh.2026.1816630)
Supplement: Supplementary file 3 [file Data_Sheet_3.PDF]

Table 3. Focus group interview guide: categories, example quotes, and inductive themes

**Interview guide questions**	Example quotations	Main themes/subthemes
	**Category 1: Pathways and Motives**	
**How did you hear about this study?**	PwMS: I became aware of the study through the DMSG Cologne newsletter and I thought it sounded interesting. I always think it's good when you can do further research somehow.	***Study awareness through German MS Society channels***<br>Couples learned about the study through German MS Society channels (newsletter and self-help groups) and by word of mouth.
**What were your reasons for taking part?**	P: The interest is simply ... that at the beginning you naturally have a lot of questions about how to deal with such an illness. Especially when you're young, you naturally have a few questions that don't just concern you for 10 years, but for 40 or 50 years, and you want to prepare yourself for that as long as you can.	***Participant motivations: Research contribution and future planning***<br>Participants cited a range of motivations for participation, including<br>(a) supporting research,<br>(b) the digital format,<br>(c) addressing gaps in prior counseling,<br>(d) focusing on the "couple perspective,"<br>(e) general openness and curiosity, and<br>(f) preparation for future challenges.
	**Category 2: Offers and Invitations**	
**Do you imagine that there would be general demand for such counseling services?**	P: ... I also think the topic is really important and I immediately remembered the time, about 20 years ago, when the symptoms started and then the diagnosis came. It was all incredibly stressful for both of us as individuals and as a couple and I can imagine that support, coaching, some form of intervention can be incredibly beneficial, especially in this phase.	***Perceived needs and the demand for couple-oriented counseling***<br>Participants generally affirmed the demand for such counseling services, highlighting needs such as<br>(a) support with future-related fears,<br>(b) early-stage guidance,<br>(c) peer exchange with other couples,<br>(d) sharing of knowledge, and<br>(e) experiences and fostering communication within the couple.
**Should counseling sessions be offered to couples living with MS? Would there be any fears to address?**	PwMS: ... So my fear would be that the more informed my husband is about the situation, the more fears he might get that he doesn't have because he doesn't have the knowledge.	***Potential emotional barriers***<br>While participants supported the idea of counseling sessions for couples living with MS, they also noted potential emotional barriers such as a tendency to avoid thinking about the future and the fear of gaining distressing knowledge.

**Category 3: Wishes, Orders, and Goals**		
**What wishes and orders would you present—in advance—and what goals would you set?**	PwMS: ... many people find it difficult to talk to their partner about things, about limitations that they have, things that happen, help that they need or that they want or don’t want, and simply to open up this space, to get the security to start this conversation with each other at all, so that silence doesn’t creep in!<br><br>PwMS: ... I use you as a role, the partner as a co-observer of the illness—“Have you noticed that too? Do I now have more word-finding problems or less?” ... and that is of course also, I think, to become aware again of what roles these are and does the partner feel comfortable with the roles or what do you need to perhaps fill the roles well and also to give the partner the space to say what is bothering him or her in any case.	***Strengthen the partnership***<br>Support in creating a balanced and empowered relationship, particularly by helping couples manage demands, boundaries, and role diffusion.<br><br>***Foster communication***<br>Provide space and guidance for open, honest discussions, including sensitive or “unattractive” topics such as incontinence.<br><br>***Engage both partners***<br>Ensure both individuals are actively involved in the advisory process.<br><br>***Professional moderation***<br>Offer high-quality moderation to avoid routine or ineffective (“business as usual”) sessions and to navigate complex dynamics effectively.
**Category 4: Content**		
**What disease-specific topics should such sessions cover?**	PwMS: In a proposal, it would probably be good to address the unattractive topics directly as well, because it can be difficult to start a conversation—for example, about incontinence.	***Disease-related topic setting***<br>Sessions should cover key MS-specific topics such as<br>(a) fatigue,<br>(b) immobility,<br>(c) incontinence,<br>(d) sexuality and intimacy, and<br>(e) the desire to have children, addressing both the practical and emotional aspects of these challenges.
**What relationship-centered topics should such sessions cover?**	PwMS: I think, especially at our age, there’s a lot of planning for the future, what’s coming up, children yes/no, can you manage it, can’t you manage it or something else, what could it look like in the future, if you’re looking for	***Relationship-related topic setting***<br>Sessions should address relationship-centered topics such as<br>(a) the role of partners,<br>(b) navigating the balance between giving and accepting help,

	an apartment, do you have to make sure you have an elevator or not. So somehow all of these issues keep you busy and I think it's always important that relatives have their own space.<br><br>PwMS: I would also appreciate it if it were a bit more resource-oriented—so not just having a room and saying, "Now we're doing a seminar, now we're doing counseling, and now we're putting all the problems on the table"—but really looking at what strengths already exist in the relationship. (…)	(c) distinguishing between illness-related and relationship issues,<br>(d) managing expectations,<br>(e) improving communication,<br>(f) fostering self-worth within the relationship, and<br>(g) planning for the future together as a couple.
**What other topics would you find useful to cover in couple-oriented counseling?**	PwMS: (…) Does it make sense to take out a 30-year loan, like some of our friends might do for an apartment or a house or something, when you don't even know if you'll be able to pay it back?	***Practical life topic setting***<br>In addition, sessions should cover practical life topics including<br>(a) housing and living arrangements,<br>(b) effective knowledge management,<br>(c) engaging in sports and nutrition as a couple,<br>(d) navigating work and pension issues, and<br>(e) understanding care needs, degrees of care, and disability classifications.
**Category 5: Organization and Delivery Formats**		
**How should such sessions be designed in terms of time?**	PwMS: I'm "Team Weekend," of course. I think it's really great when you can still chat privately in the evening over dinner or something.<br><br>PwMS: So I would actually be in favor of once a month, because you might want to bring something with you until the next meeting, perhaps in terms of topics or a bit of observation of what the others have said.<br><br>P: Especially with a topic like this, I believe that you create a completely different	***Format preferences: "Team Weekend" vs. "Team Once-a-Month"***<br>There was a "Team Weekend," perspective favoring sessions organized as a compact weekend event and a "Team Once-a-Month" perspective favoring recurring series held once a month (up to six times) on weekdays from 5 to 7 PM.<br><br>Some participants preferred digital formats, others preferred in-person events, and some suggested a hybrid format.<br><br>It is important that the venues ensure accessibility for all participants.

	relationship than you do via the screen—that in any case.	
**How should such sessions be designed with regard to the number of participants?**	P: (…) a no-go is when it's such a huge group and you somehow never get a chance to speak because 16 people are sitting there.	***Preferred group size varies by delivery format***<br>Sessions could be planned with group sizes of 4–5 couples for digital sessions and 5–10 couples for in-person formats.
**Category 6: Objectives and Target Groups**		
**For what reason, and for which couples living with MS, would counseling sessions be particularly important?**	P: ... I think the first step is, of course, a recent diagnosis, so that they can get to grips with the subject, because the uncertainty is particularly great at the beginning. If you've already spent a few years together and have the diagnosis, then it's already settled in a bit. Of course, there are always fears, uncertainties or other things or new topics such as wanting to have children or something like that, but especially at the beginning there is still this great unknown.<br><br>PwMS: I also think it's good that people are even thinking about offering something for couples. That's why we were quickly enthusiastic about it. I just hope that a format is developed from this quickly and that an offer is created for newly diagnosed individuals, so that they have an easier time dealing with the illness than we all did.	***Priority groups for counseling: Life stage and context considerations***<br>(a) Couples newly confronted with the diagnosis who are dealing with uncertainty and fear ("that great unknown")<br>(b) Younger couples (under 30) in the early stages of establishing life plans<br>(c) Couples with children<br>(d) New partnerships<br><br>***Supporting couples through role strain and emotional adjustment***<br>Sessions should support couples as they face tension or imbalance in roles and emotional disconnects during the adjustment process.<br><br>***Learning and support through mentorship and peer exchange***<br>Sessions offer opportunities for growth through professional guidance and peer connection. Those who have lived with MS for longer periods can share coping strategies, emotional insights, and encouragement—especially valuable for newly diagnosed or struggling couples.<br><br>***Flexible, participant-oriented session formats***<br>Participants support flexible session formats in which the group helps shape the topics, ensuring relevance and engagement for all involved.
**Category 7: Attractiveness Features**		
**What attractive features can you suggest? (title, type of invitation,**	PwMS: I'll be honest: when I get it by email, I... I'm on my cell phone anyway, that's the damage of the generation, and then I look at my emails and then I scroll through them at	***Reaching couples through diverse channels***<br>Key outreach features include the use of<br>(a) printed flyers,<br>(b) digital media such as Instagram and email,

**advertising channels/areas)**	that moment and then I just discover low-threshold things where I can say "Okay, I think that's exciting" and then maybe a week later I think about it and then I join in.<br><br>PwMS: And there must also be digital offers ... There are simply many people affected who don't live in Cologne or in a larger city and who don't have the opportunity to travel somewhere at half past seven in the evening.	(c) personal and professional networks, and<br>(d) medical channels such as neurology practices and clinical MS centers for distributing invitations.<br><br>Proposed titles: "Couple Time," "Healthy Partnership with MS," and "Together with MS."
	**Category 8: Design and Structure**	
**How could such sessions be designed? What methods, formats, and techniques would you find helpful in dealing with these topics?**	P: PowerPoint usually makes the first impression on me. It's a bit like an info flyer: for a first summary basic knowledge, okay, and when it goes further, it's not as conclusively helpful as personal interaction.	***Flexible and interactive session design***<br>Sessions should be designed with a flexible and interactive structure, combining large group sessions, small group discussions, and individual conversations with each couple.<br><br>Sessions should include multiple themed rooms, use techniques such as role plays to encourage perspective shifts, follow a clear and recognizable concept, and remain open to participant-driven topics collected at the beginning.
**What qualities and skills should the ideal moderator have?**	PwMS: Humor is important ... so I would actually prefer to have someone who also has MS, and preferably I would like to have a tandem. The best thing would be if it was a couple where one of them has MS. That would be the icing on the cake for me.<br><br>PwMS: ... also the goal of moving the couple forward together ... he has to get people to communicate.<br><br>PwMS: So from my point of view, the most important position ... what should he bring with him? Well, definitely background	***The most important position: Attributes of an ideal moderator***<br>Moderators should ideally be couples with one partner living with MS, to bring empathy and lived experience.<br><br>Moderation should preferably be handled by a team to ensure diverse perspectives and attentive, sensitive facilitation through the presence of "many eyes and ears."<br><br>Each moderator should be an effective communicator who fosters meaningful dialogue, brings humor to create a relaxed atmosphere, and skillfully guides group discussions.<br><br>The moderator should also possess in-depth knowledge of MS, demonstrate emotional intelligence by meeting participants where they are and handling

	knowledge of MS and perhaps a bit more in-depth knowledge.<br><br>P: For me, it would actually be someone who is not so rigid, because the topic itself is already... it's an illness... but who simply deals with it openly.	sensitive topics with care, and be capable of balancing the roles of moderator, supporter, and researcher.
**Category 9: Benefits**		
**Assuming you took part in such a session, what would need to happen for it to be a "useful" event in retrospect?**	PwMS: If I imagine the seminar had been at the beginning of the illness, then I would definitely have noticed in a positive sense that I would be a little less depressed and have fewer worries and fears about the future.<br><br>P: I'm already realizing that I've learned a bit more about my wife in this round of talks that I didn't know before.<br><br>PwMS: I think it's ultimately about connection, strengthening connections with each other, and perhaps also building new connections within the group.<br><br>P: Thinking back to the place and incorporating it into everyday life ... I'm always someone who likes to pass it on. So, I would probably notice if I told others about it and tried to pass on tips. ... But having a toolbox at the end—in quotation marks—with strategies and resources and things that you already do well and that can help you to simply keep this in mind. (...)	***Practical impact***<br>Sessions should provide a useful toolbox of strategies and resources which couples can integrate into their daily lives, leading to lasting behavioral changes and continued reference to the content.<br><br>***Relationship growth***<br>Sessions should help strengthen the connection within the couple and cultivate new perspectives, enabling more open communication and shared understanding.<br><br>***Emotional benefit***<br>Participants should experience emotional relief, with reduced stress and fewer worries about the future.<br><br>***Social and informational exchange***<br>Sessions should facilitate connections with other couples and offer practical tips and insights for everyday challenges, including dealing with authorities.

Note: MS = multiple sclerosis; P = partner; PwMS = person with multiple sclerosis.
